# Temperature variations in pharmaceutical storage facilities and knowledge, attitudes, and practices of personnel on proper storage conditions for medicines in southern Malawi

**DOI:** 10.3389/fpubh.2023.1209903

**Published:** 2023-09-22

**Authors:** Felix Khuluza, Francis Kachidza Chiumia, Happy Magwaza Nyirongo, Chifundo Kateka, Raphael Abbuh Hosea, Westonie Mkwate

**Affiliations:** Department of Pharmacy, School of Life Sciences and Health Professions, Kamuzu University of Health Sciences, Blantyre, Malawi

**Keywords:** pharmaceutical storage conditions, temperature variations, mean kinetic temperature, Malawi, medicine storage conditions, storage in-a-box (SIAB), pharmacy/medicine storage areas

## Abstract

**Objective:**

We assessed the temperature variations in pharmacies and medicine storage areas in southern Malawi and conducted a knowledge, attitude and practices survey for personnel who manage medicine stores in various health facilities.

**Methods:**

This was a longitudinal study design that used installed Tempmate^®^ thermometers in 27 selected health facilities to record temperatures every 15 min for a period of 9 months. In addition, a questionnaire was used to assess the knowledge, attitude, and practices regarding good pharmaceutical storage. Observations were also made on the storage structures of the facilities and compared with the mean kinetic temperature.

**Results:**

Storage temperature ranged from 13.8°C to 42°C with mean kinetic temperature (MKT) being 25.3°C (95% CI 24.4–26.2°C). Mean temperature for public facilities was lower (23.8°C) than the faith-based facilities (25.2°C) and private facilities (26.6°C). In terms of level of health care, lower temperatures were recorded in facilities offering tertiary level of care as compared to secondary and primary care facilities, *p* < 0.001. For the type of storage facilities, storage-in-a-box unit (SIAB) presented lower temperatures than ordinary storage areas (non-SIAB), *p* < 0.001. Majority of health workers (69%) had good knowledge on proper storage conditions. Air conditioners and thermometers were available in 88.4 and 76.9% of the facilities, respectively. However, few facilities utilized the air conditioners due to electricity problems. About 46.15% of the participants were able to correctly record temperatures (at least twice a day) for the storage facilities, 23.07% did not properly record while 30.77% of the personnel did not keep temperature records at all. Limited storage space was among the challenges that facilities encounter to maintain proper storage conditions.

**Conclusion:**

Despite having the necessary knowledge on proper storage conditions, the pharmacy personnel failed to adhere to good pharmaceutical storage practices due to resource limitations. There is a need for stakeholder interventions such as increasing budget allocation to address the challenges faced by the health facilities.

## Background

Maintaining appropriate storage conditions is one of the fundamental principles of the World Health Organization (WHO) good storage practices for pharmaceutical products (1). Environmental factors such as temperature and humidity have direct impact on the stability of pharmaceutical products and may lead to deterioration before the products reach their expiry date (2). In some cases, the effects of harsh conditions may be noticeable by alterations in physical characteristics of the medicine formulation such as color, texture and tensile strength (3, 4). On the other hand, chemical degradation which usually affects the composition of the medicines may require laboratory analysis for content of active ingredients and presence of metabolites or impurities to be detected (5). In either case, any form of medicine deterioration has potential to cause harmful patient outcomes such as loss of efficacy and adverse drug reactions (6).

High temperatures accelerates mechanisms of reaction for medicine degradation such as oxidation and hydrolysis, which affects the potency of medicine and also yield toxic products (7). For instance, hydrolytic degradation of isoniazid under high temperatures forms hydrazine which has shown significant genotoxicity and carcinogenicity in animal studies (8). High temperatures also facilitate conversion of enalapril to enalaprilat and diketopiperazine which affects the bioavailability of the drug and reduce efficacy (9). Theoretically, the rate constant of chemical degradation reaction (*K*) will increase due to increase in temperature (*T*) as stated by the Arrhenius equation: $=A^{\left(-Ea/RT\right)}$ , where *A* is the pre-exponential or frequency factor, *E_a_* is the activation energy of the reaction and *R* is the ideal gas constant (10).

Usually, medicine manufacturers provide guidance on the recommended storage conditions for the products. However, temperature excursions are sometimes inevitable especially in resource limited facilities where temperature control mechanisms for the storage rooms are not sufficient (11). As such, the mean kinetic temperature (MKT) provides a better estimate of the expected level of degradation that may have occurred under various temperature conditions (12). The MKT is defined as the “*a single derived temperature, that if maintained over a defined period of time, affords the same thermal challenge to a drug product as would be experienced over a range of both higher and lower temperatures for an equivalent defined period*” (13). The MKT is calculated based on the degradation kinetics as modeled by the Arrhenius equation using the formula:


$$T_{k}=\frac{E_{a}/R}{-\ln\left(\frac{e^{\left(-E_{a}/RT_{1}\right)}+e^{\left(-E_{a}/RT_{2}\right)}+\ldots +e^{\left(-E_{a}/RT_{n}\right)}}{n}\right)}$$

where T_k_ is the mean kinetic temperature, E_a_ is the activation energy, R is the universal gas constant, T is the average temperature at a particular time, and n is the number of temperature recordings for the defined duration (12).

An estimate of MKT can be calculated using the climatic data for a particular region. Based on these estimates, WHO classifies countries into four climatic zones: temperate (zone I); subtropical (zone II); hot and dry (zone III); hot and humid (zone IV). Long-term stability studies for the climatic zones are carried out under temperatures and relative humidities (RH) of 21°C/45% RH (Zone I), 25°C/60% RH (zone II), 30°C/35% RH (Zone III), and 30°C/70% RH (zone IV) respectively (14). Malawi is classified under the subtropical climate (15). The United States Pharmacopeia (USP), recommends storage of ordinary dosage forms such as oral tablets and capsules under a maximum of MKT 25°C with allowable excursions between 15°C and 30°C (16).

Daily mean temperatures for Malawi derived from open air conditions range from 13°C–35°C and are extremely variable across the districts, with some areas reaching up to 42°C (17). Temperature conditions continue to increase due to global warming putting many countries at a risk of climate crisis which is a public health concern (18). In low- to middle-income countries such as Malawi, there is insufficient mechanisms to control temperatures within the pharmacy and medicine storage areas. This is also further influenced by insufficient electricity supply and high cost of fuel (18). However, no studies have been done to assess the influence of atmospheric conditions on medicine store temperatures. We therefore assessed the temperature variations in medicine stores in southern Malawi and conducted a knowledge, attitude and practices survey for personnel who manage medicine stores in various health facilities.

## Methodology

### Study design/type of research

This was a longitudinal research study design which was conducted at different private and public pharmacies. We installed Tempmate^®^ thermometers in selected health facilities. In addition, we interviewed pharmacy personnel using structured questionnaire on their knowledge, attitude and practices regarding pharmaceutical storage practices.

Tempmate^®^ thermometers were used to collect the temperature and humidity. Tempmate^®^ is a type of thermometers capable of recording temperature every 15 min and deciphered from a computer. We decoded data of temperature variation for nine consecutive months in all facilities.

On Knowledge, Attitude and Practices (KAP), questionnaires were administered to personnel who were manning the pharmacy or drug storage areas to assess how different drug formulations are being stored in terms of temperature ranges. In addition, we were observing the storage practices by inquiring the availability of temperature and humidity monitoring devices, apart from the Tempmate^®^ logger that we installed. This was based on the understanding that each facility is supposed to have the temperature and humidity monitoring devices, while the Tempmate^®^ was placed in the facilities based on our study as a comparator.

We also observed if the temperature and humidity monitoring systems were well calibrated and working perfectly. On the KAP, we also assessed the alternatives used in case of emergencies, i.e., electricity blackouts. The participant’s knowledge was assessed on how they address the challenges encountered in maintaining proper storage conditions.

### Study place

The study was conducted in three selected districts of the southern region of Malawi namely Zomba, Machinga and Nsanje. We placed the Tempmate data loggers in public, faith-based and private pharmacies and drugstores. Details on the how the sites were selected have been described in another publication (19). In summary, we installed Tempmate® in 11 faith-based not for profit, 16 public and two private health facilities. Two public primary level facilities were removed from the study at the analysis stage due to loss of data collection tool and incompleteness of data as a result of technical errors (premature stops of Tempmate^®^ leading to missing values), so in the end a total 27 facilities remained in the analysis (11 faith-based not for profit, 14 public and two private health facilities, i.e., 21 primary, 5 secondary and one tertiary level health-care facility).

### Data collection

Temperature conditions in pharmacies and medicine storage areas were recorded automatically for every 15 min from September 2021 to May 2022, using the Tempmate® data loggers. This included both two seasons of Malawi: 3 months of the cool dry season and 6 months of the hot wet season. A longer duration was considered for the hot-wet season as it is non-conducive for pharmaceutical storage and usually longer. The data loggers were set up in a way that could only be stopped using a software with a password to prevent interruptions or data manipulation. To assess the knowledge, attitudes, and practices of pharmacy personnel on the storage of pharmaceutical products, we administered a questionnaire to personnel who were manning the medicine stores for the study health facilities. The questionnaire included participant’s demographics such as participant’s sex, age, profession, and qualification; availability of temperature control and monitoring tools; and compliance to recommended storage practices. This was supplemented by in-depth interviews that were semi-structured to explore the storage challenges that are faced by the personnel. The interviews were recorded using a digital voice recorder and took an average of 20 min. Both the questionnaire and semi-structured interview guide were pre-tested before administering to our participants.

### Data management and analysis

All quantitative data were entered in Microsoft excel and analyzed using STATA version 14.1. Temperatures were summarized as arithmetic means, mean kinetic temperatures and ranges, and were presented as tables, graphs and figures. Histogram plots were used to check for normality of data distribution. We used T-test to compare means between two characteristics of medicine storage facility. Where the medicine storage facility was categorized into more than two groups, ANOVA was used to compare the differences. Further, a Bonferroni test for multiple comparison was used as a *post hoc* test subsequent to ANOVA. A *p* value of <0.05 was considered as significant for all tests. For the qualitative data, voice recordings were transcribed and analyzed by thematic approach. To avoid bias, the analysis was performed by two investigators independently. Where the interview used Chichewa language, the content was translated verbatim into English by the investigators who were all fluent in both languages. The transcripts were read for multiple time for data familiarization and key themes were derived manually from the text.

### Ethics approval and consent to participate

The study protocol was reviewed and approved by the College of Medicine Research and Ethics Committee under study number P.10/21/3447. In addition, permission was sought from the hospital director or directorates of health and social services in Zomba, Machinga and Nsanje. There was no patient intervention that was done at any stage of the study. All participant data were coded, and no names and any identifiers were used. Further, for those participants that were interviewed, they were provided with written participant information sheet and an oral explanation about the study before signing the consent forms. The participants’ consent included the use of voice recorder.

## Results

### Temperature conditions in facilities during study duration

**Figure 1:**
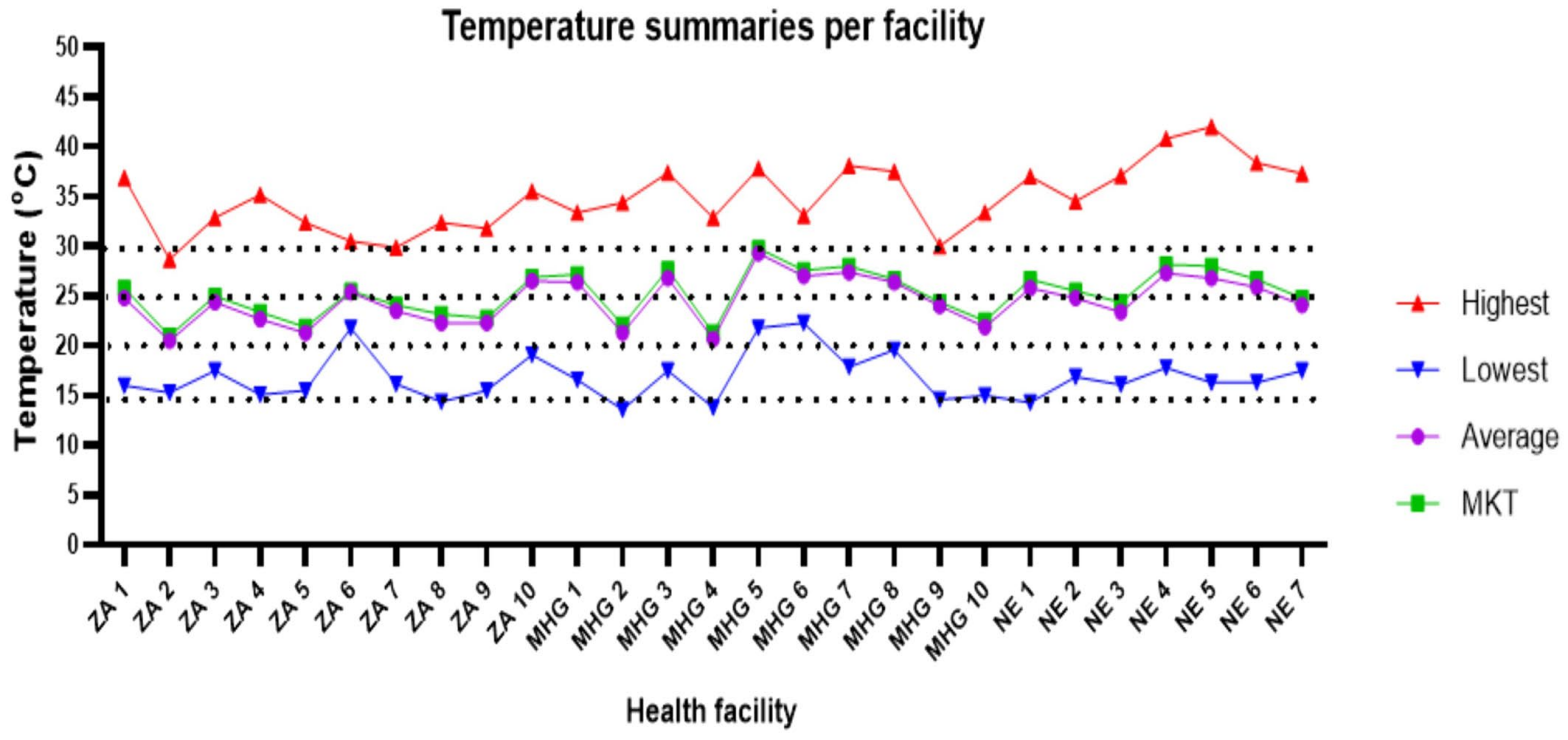
Summaries for temperatures recorded during the study period. ZA, Zomba; MHG, Machinga; NE, Nsanje.

We collected daily temperature conditions for every 15 min for duration of 9 months from September 2021 – May 2022. For this period, temperatures range was 13.8°C–42°C. Highest temperatures in most of the facilities (88.9%, *n* = 24) were above 30°C and only 11.1% (*n* = 3) of the facilities registered a highest temperature below 30°C for the entire study period. Only one (3.7%) facility registered a minimum temperature below 15°C during the study period. The mean of average temperatures was 24.6°C (95% CI 23.7–25.5°C), while for mean kinetic temperature (MKT) was 25.3°C (95% CI 24.4–26.2°C). MKTs were > 20°C ≤ 25°C for 48% (n = 13) of the facilities, and > 25°C ≤ 30°C for 52% (*n* = 14) of the facilities (Figure 1). MKTs were higher than the average temperatures for the health facilities, *p* < 0.001.

**Figure 2:**
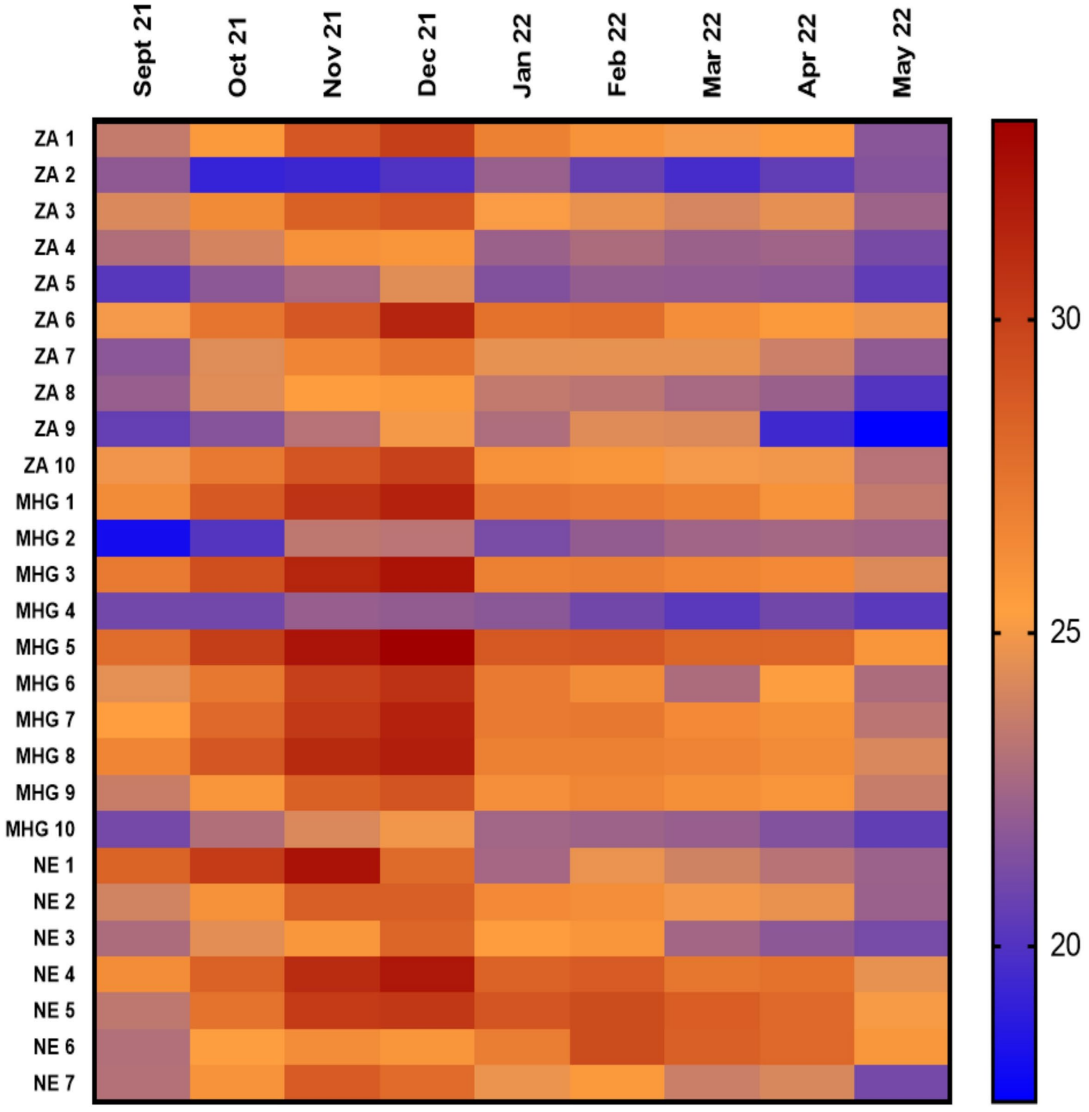
Shows the mean kinetic temperature for the 9 month period (horizontal line shows the months while the vertical line are the facilities). ZA, Zomba; MHG, Machinga; NE, Nsanje.

Monthly average temperature for most of the facilities were between 25°C–30°C in the months of January, February, March, April and September (Supplementary Figures S1A–C). Around November and December, 37% (*n* = 10) of facilities had average temperatures above 30°C. Thereafter, temperatures gradually decreased. By May, average temperatures for 88.9% (*n* = 24) of the facilities were below 25°C except for only one facility in Machinga and two facilities in Nsanje (Figure 2).

**Figure 3:**
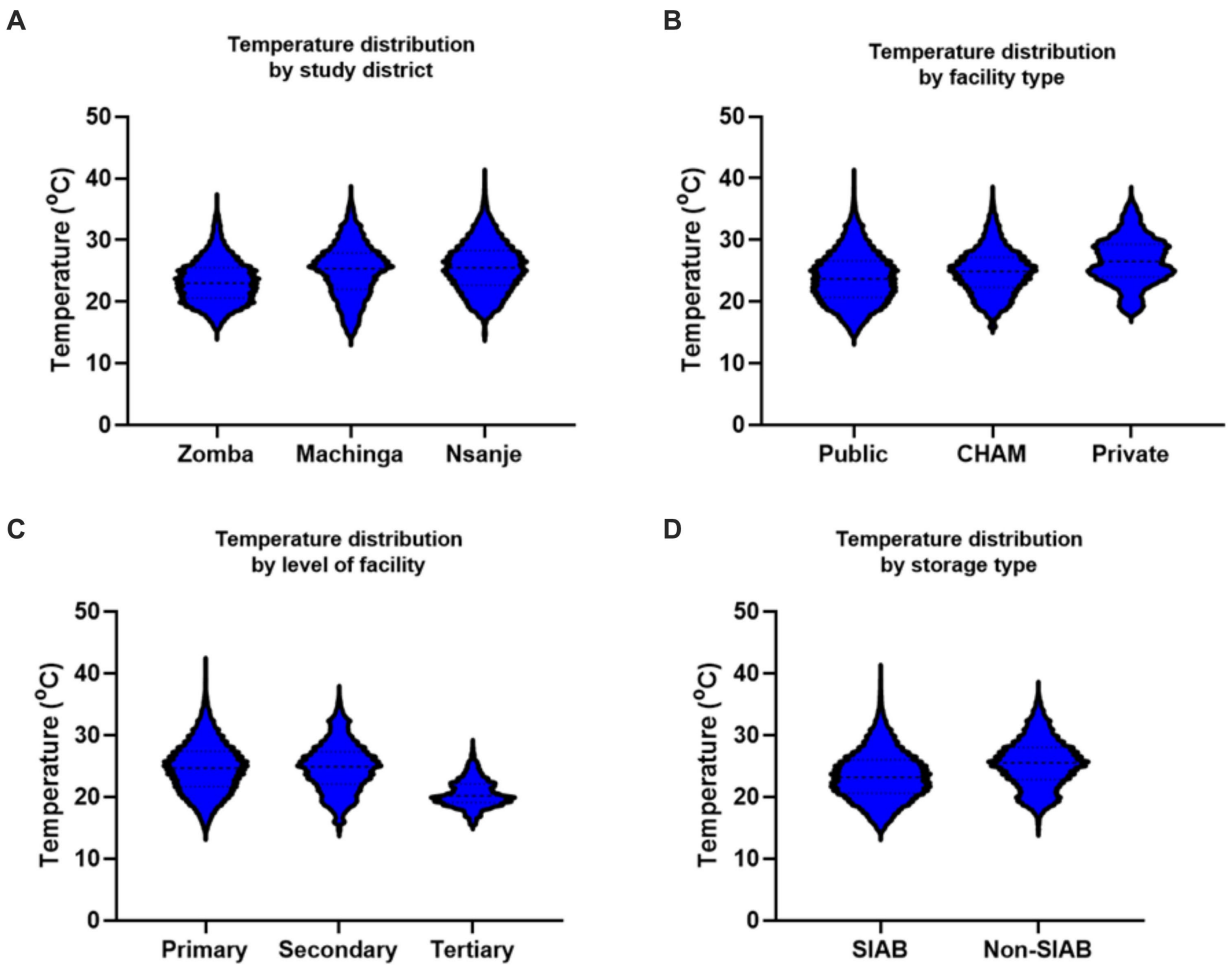
**(A–D)** Showing temperature variations among various parameters. CHAM, Christian Health Association of Malawi; SIAB, Storage in a Box Unit.

Temperature distribution was further compared based on characteristics of the health facilities. Zomba facilities had lower temperatures as compared to Machinga, which was lower than Nsanje, *p* < 0.001. The mean temperatures for facilities in Zomba, Machinga and Nsanje districts were 23.1°C (Interquartile range (IQR) 20.5–25.3°C), 25.0°C (IQR 22–27.9°C) and 25.5°C (IQR 22.4–28.2°C) respectively (Figure 3A). Health facilities in Malawi are owned by government (public facilities), faith-based organizations or private individuals. Public facilities registered lower temperatures as compared to faith-based facilities, which were lower than temperature in private facilities, *p* < 0.001. Mean temperature for public facilities was 23.8°C (IQR 20.6–26.5°C). For the faith-based facilities, the mean temperature was 25.2°C (IQR 22.7–27.5°C), while for private facilities it was 26.6°C (IQR 24.0–29.3°C) as can be seen in Figure 3B. In terms of level of health care, there were lower temperatures in facilities offering tertiary level of care as compared to secondary care, which was lower than primary care facilities, *p* < 0.001. Mean temperatures for primary, secondary and tertiary level facilities were 24.7°C (IQR 21.7–27.4°C), 24.8°C (IQR 22.1–27.3°C), and 20.5°C (IQR 19.1–22.1°C) (Figure 3C).

For the type of storage facilities, storage-in-a-box unit (SIAB) presented lower temperatures than ordinary Pharmacies and medicine storage areas (non-SIAB), *p* < 0.001. Mean kinetic temperature for the SIAB facilities was 23.5°C (IQR 20.6°C–26.1°C) while for the non-SIAB facilities was 25.4°C (IQR 22.8°C – 28.0°C), Figure 3D.

### Knowledge and practice of pharmacy personnel in health facilities

#### Demographics of personnel

A total of 26 personnel from the 27 facilities participated in the survey of which 76.9% (*n* = 20) were male and 23.1% (*n* = 6), were female. Among the facilities, 57.7% of the pharmacies were manned by pharmacy professionals while 11.5% were manned by medical assistants at the time of the survey. The other 30.8% of the facility pharmacies were manned by non-skilled personnel (Pharmacy attendants). The pharmacy attendants had a highest qualification of either junior high school (JCE) or senior high school (MSCE) certificate (Table 1). For the professional pharmacy personnel, they were holders of a professional certificate (*n* = 7), diploma (*n* = 4), first degree (*n* = 3) and post graduate degree (*n* = 1) in pharmacy. Thus, there were only 4 registered pharmacists who were serving at secondary and tertiary level hospitals and private pharmacies.

**Table 1:** Demographic characteristics of pharmacy personnel.

Variable	Characteristic	No. of participants	Percentage (%)
Sex	Male	20	76.92
	Female	6	23.08
Age group (Years)	18–30	11	42.31
	31–45	10	38.46
	46 and above	2	7.69
	Unknown	3	11.54
Experience (Years)	0–5	12	46.15
	6–15	3	11.54
	16 and above	8	30.77
	Unknown	3	11.54
Qualification	JCE	2	7.69
	MSCE	5	19.23
	Certificate in Medicine	3	11.54
	Certificate in Pharmacy	7	26.92
	Diploma in Pharmacy	4	15.38
	Degree in Pharmacy	3	11.54
	Postgraduate degree	1	3.85
	Unknown	1	3.85
Cadre	Medical assistant	3	11.54
	Pharmacy attendant	8	30.77
	Pharmacy assistant	7	26.92
	Pharmacy technician	4	15.38
	Pharmacist	4	15.38

### Knowledge, attitude and practice of pharmacy personnel on storage of pharmaceutical products

Participants were assessed on the knowledge of required storage conditions for various pharmaceutical products. About 69% of the participants had knowledge of the general required storage conditions for oral solid dosage forms. Twenty seven percent gave wrong responses and 4% indicated that they did not know the correct conditions (Table 2). For products that require refrigeration, 42% gave the correct responses, 35% gave wrong responses and 23% indicated that they did not know the required conditions. For topical semi-solid dosage forms, 58% showed knowledge of the required storage conditions, 35% gave wrong responses and 8% indicated that they did know the required storage conditions. Participants’ attitudes were assessed by their perception on the consequences of deviating from the correct storage conditions for pharmaceutical products. All the participants (100%) provided correct responses.

**Table 2:** Showing knowledge, attitudes and practices of personnel on storage of various pharmaceutical products.

Assessed item	Response	Percentage
Storage of oral solid dosage forms	Correct	69.23%
	Incorrect	26.92%
	Do not know	3.85%
Storage of refrigerated products	Correct	42.31%
	Incorrect	34.62%
	Do not know	23.08%
Storage of topical semi-solid dosage forms	Correct	57.69%
	Incorrect	34.62%
	Do not know	7.69%
Effects of unfavorable temperature on pharmaceutical product	Correct	100%
	Incorrect	0%
	Do not know	0%
Availability of air conditioners	Yes	88.46%
	No	11.54%
Availability of alternative power source, e.g., power gen set	Yes and is functional	38.46%
	Yes but not functional	26.92%
	No	34.62%
Availability of room thermometers	Yes	76.92%
	No	23.08%
Thermometers functional	Yes	65.38%
	No	34.62%
Frequency of recording stores temperature	Once a day	7.69%
	Twice a day	46.15%
	Once a week	7.69%
	Once a month	7.69%
	Do not record	30.77%
Control of temperature during transportation	Cooler boxes	69.23%
	Air conditioned vehicles	0%
	No control	30.77%

Storage practices were assessed by the availability and usability of the tools that support the maintenance of appropriate conditions in medicine stores (Table 2). Air conditioners and thermometers were available in 88.4 and 76.9% of the facilities, respectively. However, only 38.5% of these facilities had functional back-up energy to power the air conditioners in times of electricity black outs. In addition, only 65.38% of the available thermometers in these facilities were functional. Furthermore, only 46.15% of the participants were able to correctly record temperatures (at least twice a day) for the storage facilities and 30.8% of the personnel did not keep temperature records at all. During transportation of medicines among facilities, 69.2% of the participants used cooler boxes, and 30.77% did not use any temperature control mechanism.

### Challenges encountered by health facilities on pharmaceutical storage

Limited storage space was among the challenges that facilities encounter to maintain proper storage conditions. In some facilities, pharmaceutical products were shifted to temporary rooms that were not properly designed and not suitable for storage purpose due to limited space in the main pharmacies and medicine stores. These temporary rooms did not have proper mechanisms to control the temperature conditions.

“*The room is just too small to be used for the purpose. As you can see the cartons are almost up to the roof and there is no ceiling*,” participant # 16.

Participants also reiterated about intermittent power outages, which was one of the main causes of failure to control temperature in the pharmacies and medicine stores. The problem was worse in Nsanje district especially during rainy seasons as the place is usually hit by floods and thereby causing much more damage to infrastructure. Moreover, most of the roads are rendered impassable and thereby affecting maintenance works for the energy infrastructure. Even though some facilities had alternative source of energy such as generators, they failed to utilize them during electricity blackouts due to lack of fuel or maintenance of the devices.

“*We have intermittent electricity supply here more especially in rain season. To make it worse we do not even manage to run the generator due to finances*” participant #1.

“*The problem we face is persistent blackouts more especially in rain season where we do stay for about 3 weeks before the electricity is back again, and you know Nsanje has high temperatures, and we cannot use the generator through the day. We are only allowed to use it for a few hours. It also happens that in such times the generator is not functioning. When there is no electricity here temperatures can go up to 49 degrees Celsius in the storage rooms*” participant # 6.

“*We have electricity problem for more than 6 months now*” participant # 13.

The other major challenge was lack of mechanisms or devices for control and monitoring of temperatures in the storage rooms. Although most of the facilities had air conditioners, most of them were not being used due to financial constraints. Moreover, some of the air conditioners developed faults and no efforts to maintain them were initiated.

“*In my stores, we are running out of gas in the air conditioners and at times it becomes hotter such that the temperatures can exceed 30°C such that we fail to maintain consistent temperatures” participant* # 4.

Some facilities were also failing to keep records of the temperatures in the rooms as they did not have proper thermometers.

“*We do not have thermometers. We only depend on the thermometers embedded on the refrigerators and the ones displayed on the air conditioners*” participant #14.

On implication of adverse impact of not adhering to proper storage, almost all respondent agreed that they experienced or received reports about medicine degradation within the health facilities. Some further reiterated about unfavorable clinical outcomes such as loss of efficacy due to use of medicines which were stored under poor conditions in their facilities. Some of the stories shared were as mentioned by the following participants:

“*Sometime due to high temperatures even the suppositories do melt. We have had such incidences before, and we no longer use such drugs*” participant #6.

“*The time we were having cyclone Ana we issued oxytocin, but we were given feedback that the drug wasn’t working. It also happened with anesthetic, lignocaine, such that when it was given to the patient the pain was still there*” participant #16.

#### Impact of electricity supply on pharmaceutical storage

Intermittent power supply and high running cost of air-conditioners were the common problems among facilities that recorded higher temperatures during the study period. Although some facilities had functional air-conditioners, they stated that they could not always use them due to high electricity bills incurred when using them. In addition, facilities thought it was even more costly to use generators whenever electricity supply was interrupted. Thus, very few facilities made use of the fuel powered electricity generators as an alternative energy source.

Participants were able to appreciate the negative impact of non-conducive storage conditions through their own experiences. These included visually notable deterioration of medicines and reported loss of efficacy of medicines that were exposed to higher than recommended storage temperatures.

## Discussion

We analyzed the temperature variations in three randomly selected districts in Southern Malawi. Though Malawi is classified as subtropical (zone II) by WHO (15), there is a great temperature inter-variability. Generally, within the southern region of Malawi, Zomba records the lowest temperatures while Nsanje records the highest temperatures around the year as compared to the rest of the districts in the regions (20). In this study, we focused on assessing the temperatures in pharmacies and medicine storage rooms and the challenges faced by facilities in maintaining proper storage conditions. Our findings reveal that there was minimal control of the temperatures in medicine storage facilities. We found that the medicine stores temperatures were changing in a similar pattern as the open-air temperatures based on the geographical location of the facility and season of the year. As observed, facilities in Zomba had the lowest temperatures as compared to Machinga and Nsanje, which had the highest temperatures. The temperature variation among the districts followed the normal weather patterns of the districts with Zomba district (situated along the Zomba mountain) having its average temperature throughout the year being 20.6°C, while Machinga is 27°C and Nsanje being above 30°C (21–25) Malawi has a sub-tropical climate characterized by two main seasons: cool dry season between May and October and hot wet season between November and April (17). Mean outside temperatures are reported between 17°C and 27°C in the cool dry season while in the hot wet season vary between 25°C and 37°C (26). Coldest temperatures are experienced between May and July with frost occurring in selected parts of the country (27). In the study, we generally observed an increase of temperatures from the beginning of the hot wet season (September) and a decrease towards the start of the cool dry season (May). During the hot wet season, very few facilities maintained consistently lower than seasonal temperatures within the storage facilities, which was probably due to use of air conditioning systems.

In January and February 2022, Malawi was hit by the cyclone Ana which caused extensive property damage and massive power outages in some parts of the Central and Southern Malawi (28, 29). For instance, there was a national black out on 24th January 2022 which was sustained for more than 30 days in some health facilities in Nsanje district. As indicated in Figure 1, about three facilities experienced very high temperatures (≥30°C) during the month of February as these facilities did not also have alternative source of energy. This again is in line with observation from the participants who indicated that there was no alternative power during electricity outages. The high temperatures experienced during this time might have an adverse effects on the storage of medicines and medical supplies as most medicines are supposed to be stored under ambient temperature (15-25°C), with USP recommending up to 30°C (30).

The SIAB facilities recorded lower temperatures as compared to ordinary medicine stores. SIAB facilities were constructed by a United States Agency for International Development (USAID) project to improve quality and capacity of medicine storage facilities in Malawi. Even though this was a short-term remedy, SIAB facilities seem to offer better conditions as they contain air conditioning systems and heat reflecting paint. Even though air conditioners may occasionally be used, the SIAB are further protected with an insulated wall panel which helps to maintain the cooled temperature inside for a longer duration. Furthermore, SIAB facilities contain both inside and outside thermometers for easy temperature monitoring (31). The SIAB facilities mainly targeted public health institutions and therefore fewer faith-based health institutions benefited from the project. As observed, public health facilities recorded lower temperatures as compared to faith-based health facilities. On the other hand, no private facility contained an air conditioner in the pharmacy. As such, they recorded the highest temperatures among the health facilities. It is therefore important that the SIAB initiative should be extended to almost all faith-based to help maintain lower temperatures that will safeguard the medicines and medical supplies. This should be taken serious as 29% of primary health service delivery is provided by faith-based facilities most of whom have service-level agreement with government of Malawi (32, 33).

Availability of funds may be also one of the enablers for maintaining proper storage conditions in facilities. Generally, tertiary hospitals are most funded as compared to secondary and primary level health facilities, in that order. As noted, the tertiary level health facility was the only facility able to maintain low average temperatures (<25°C) for the entire 9-months duration. Most of the primary health care facilities were not able to use their air conditioners as they could probably not meet the high cost of running and maintaining the air conditioners using generators whenever there was electricity black-out.

Shortage of trained pharmacy personnel is also a major challenge in the health facilities. Over 30% of health facilities, mostly primary health care centers were found to be managed by non-skilled personnel commonly referred to as pharmacy attendants or drug store clerks. Task shifting is common practice in countries faced by critical shortage of health care professionals (34). A study done in Tanzania found out that more than 50% of facilities which were managed by non-pharmacy personnel were faced with critical shortages of essential medicines and did not meet the requirements for good storage practices (35). Similarly, we found that pharmacy attendants had very poor knowledge on the required storage conditions and were not able to adhere to good storage practices such as recording daily temperatures. To ensure that the quality of health services is not compromised, WHO recommends that task shifting should be continuously monitored and economically evaluated (36). Currently, pharmacy assistant training has been re-introduced with four faith-based colleges and Malawi College of Health Sciences spearheading the initiative. This training could help to solve the shortages of pharmacy personnel in the rural primary health facilities and put a stop on task shifting in the medicine storage areas and thereby improve pharmaceutical services in the rural areas (31).

Even though the perception of personnel working in medicine storage facilities is good regarding the importance of maintaining good storage conditions, majority of personnel demonstrated poor knowledge on the required temperature and conditions and did not comply to standards of good storage practice. This challenge was observed across all facilities, thus there is a need for policy shift from predominantly focussing on supply of medicines, but also incorporate good storage practices and quality assurance.

Main thematic areas regarding the challenges faced by facilities were intermittent power supply and lack of adequate resources. Malawi is among the countries with lowest electricity generation capacity. As of 2017, only about 13% of the population had access to electricity (37). With high population growth rate, the demand for electricity continues to rise leading to intermittent power supply which eventually also affects institutions that offer essential services such as health facilities (38). Availability of alternative energy source is critical for maintaining of proper storage conditions in health facilities. Much as most of the facilities have managed to source alternative energy sources such as fuel powered generators, they still face financial challenges to purchase fuel and service the devices. As noted, a substantial number of facilities were not able to utilize the generators which lead to uncontrollably increase of temperatures in storage rooms. There is a need for lobbying for government of Malawi to increase funding to health facilities so that fuel for generators should be procured without necessarily affecting other sides of health care. In addition, Malawi being blessed with sunshine, the alternative would be to install solar powered air-conditioning system in most health centers. Currently, very few health centers had solar powered installed. Solar will not only combat intermittent power shortages, but it will also reduce the cost of electricity and thereby channeling the savings to other essential medicines.

Poor pharmaceutical storage has direct impact on the quality of the products (39). High temperatures accelerate degradation mechanisms for products such as hydrolysis and oxidation (7). These types of degradation may either render the product lose efficacy or cause unexpected adverse drug reactions due to presence of toxic impurities. As described by the participants, ineffective oxytocin was noted and suspected to be caused by product degradation. A study conducted in 2019, found that 33% of health facilities with refrigerators failed to maintain required temperatures for the storage of oxytocin (2-8°C) while 42% of facilities without refrigerators failed to maintain the required storage temperatures. Furthermore, it was found that 11% of oxytocin samples from these sites contained lower than the declared amount of active ingredient (40). Even though further studies are required to investigate if the out of specification products are as a result of degradation, these results provide a serious safety signal that needs swift action.

## Conclusion

While Malawi is classified as subtropical, this study has shown that there is a great variability in temperature within Malawi. As such, there is a need to re-look into the classification and assess if it is feasible to re-classify to zone IV. This would guide pharmaceutical manufacturers to produce drugs that will withstand the temperature variations observed in this study. The study has also shown poor pharmaceutical storage practices from untrained personnel, and therefore the need for recruitment of more pharmacy personnel to manage the medicine storage areas.

## Data availability statement

All data utilized during this study are included in this article and Supplementary material. The raw datasets used and/or analyzed during the current study are available from the corresponding author on reasonable request.

## Ethics statement

The studies involving humans were approved by College of Medicine Research and Ethics Committee (https://www.kuhes.ac.mw/research-ethics/). The studies were conducted in accordance with the local legislation and institutional requirements. The participants provided their written informed consent to participate in this study.

## Author contributions

FK conceptualized, collected data, performed analysis, drafted original manuscript, revised the manuscript, supervised the work, and funding acquisition. FC collected data, performed analysis, drafted original manuscript, and revised the manuscript. HN collected and analyzed data inputs and drafted original manuscript. CK, RH, and WM conceptualized the idea, collected data, analyzed data, and drafted the manuscript. All authors contributed to the article and approved the submitted version.

## Funding

The author(s) declare financial support was received for the research, authorship, and/or publication of this article. This study was part of the European and Developing Countries Clinical Trials Partnership 2 (EDCTP2) program supported by the European Union (under grant number TMA2019CDF-2768 COPSMEDS) whose principal recipient of the grant is FK. CK, RH, and WM also received partial student funding from Kamuzu University of Health Sciences. FK is also a Consortium for Advanced Research Training in Africa (CARTA) fellow. The funders had no role in study design, data collection and analysis, decision to publish, or preparation of the manuscript.

## Conflict of interest

The authors declare that the research was conducted in the absence of any commercial or financial relationships that could be construed as a potential conflict of interest.

## Publisher’s note

All claims expressed in this article are solely those of the authors and do not necessarily represent those of their affiliated organizations, or those of the publisher, the editors and the reviewers. Any product that may be evaluated in this article, or claim that may be made by its manufacturer, is not guaranteed or endorsed by the publisher.

## Supplementary material

The Supplementary material for this article can be found online at: https://www.frontiersin.org/articles/10.3389/fpubh.2023.1209903/full#supplementary-material
