# Supplementary figures and images for: Temperature variations in pharmaceutical storage facilities and knowledge, attitudes, and practices of personnel on proper storage conditions for medicines in southern Malawi
Source: Front Public Health. 2023 Sep 22;11:1209903. doi: 10.3389/fpubh.2023.1209903 (PMC10556513; doi:10.3389/fpubh.2023.1209903)

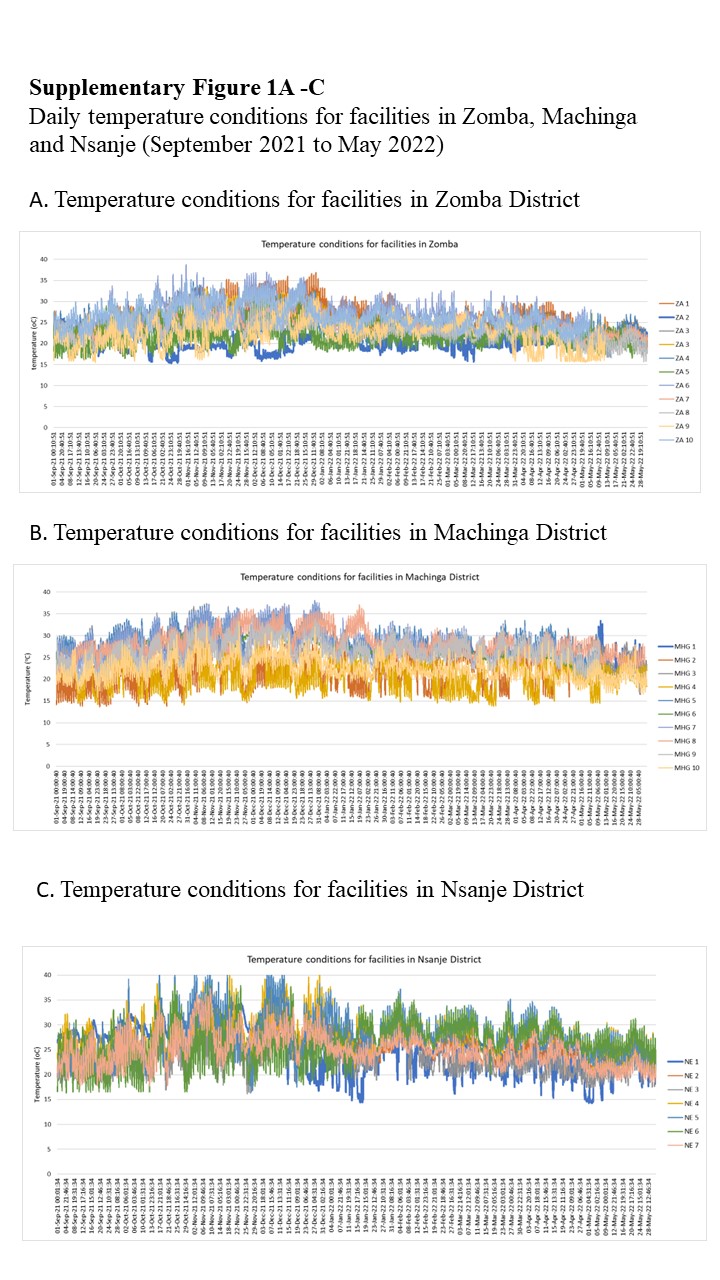

Supplement: Supplementary file 1 [file Image_1.JPEG]
