# Supplementary material for: Temperature variations in pharmaceutical storage facilities and knowledge, attitudes, and practices of personnel on proper storage conditions for medicines in southern Malawi
Source: Front Public Health. 2023 Sep 22;11:1209903. doi: 10.3389/fpubh.2023.1209903 (PMC10556513; doi:10.3389/fpubh.2023.1209903)
Supplement: Supplementary file 2 [file Data_Sheet_1.pdf]

ASSESSING KNOWLEDGE, ATTITUDE AND PRACTICES OF PHARMACY PERSONNEL ON ADHERENCE TO PROPER STORAGE CONDITIONS FOR MEDICINES IN TERMS OF TEMPERATURE VARIATION IN ZOMBA, MACHINGA AND NSANJE

Participant's code number …………… Tel…………………………….

Facility code………………………………………………………….

1. Gender

   Male …………………….

   Female……………………

2. Age…………………….
3. Working experience (years)…………………………
4. Educational level

   JCE ☐ MSCE ☐ Professional Certificate ☐ Diploma ☐ Bachelor's Degree☐

   Postgraduate Degree ☐

5. Position (e.g. Pharmacy Technician): ………………………………………………….
6. At what temperature ranges do you store the following drug formulations in your pharmacy/drugstore…

   (a) Refrigerated (Heat sensitive drugs) ……………………Don't Know ☐

   (b) Oral solid dosage forms……………………………… Don't Know ☐

   (c) Topical semi-solid dosage forms………………………. Don't Know ☐

7. Do you have air-conditioners in the pharmacy/medicine store?

   Yes ☐ No ☐

8. Do you have alternative source of energy (e.g. gen set or solar)

   Yes and is functional ☐ Yes but not functional ☐ No ☐

9. Do you have thermometers for monitoring temperature in the pharmacy/ medicine store?

   Yes ☐ No ☐

10. Are available thermometers functional?

Yes ☐ No ☐

11. How frequent do you record temperatures in the pharmacy/medicine store?

    Do not record ☐ Once a day ☐ Twice a day ☐ Once a week ☐ Once a month ☐

12. What are the effects of not adhering to the storage temperatures for medicines and medical supplies

    Don't know ☐ Nothing can happen if the medicine is before expiry ☐ Degradation ☐

13. When transporting medicines, what measures do you use for controlling temperature?

    No control ☐ Cooler boxes ☐ Air-conditioned vehicles ☐

Guide questions for semi-structured interview

1. What Challenges do you encounter in maintaining proper storage conditions?
2. How do you address the challenges encountered in maintaining proper storage conditions for medicines?
3. What quality systems do you have at the facility?
4. Share any experiences of observed negative effects as a result of storage conditions in the pharmacy/medicine store?
5. Are there any measures being established by stakeholders to improve the quality of pharmaceutical storage at the facility?
